# Offering gifts for life

**Published:** 2014

“God shows the future very rarely and for only one reason: when it is a future which was meant to be changed.”

***Paulo Coelho,***

It can be a symbol, a sign or an example. The doctors and students in medicine not only fight to prolong the life of the patients but also fight to offer quality to their lives by giving for life.

It was not for the first time in my live when I witnessed an act of normality, which, maybe, only the ones who give themselves or will give themselves to assure the health of their kind are able to do it.

Everything started from an initiative of “Elena Vacarescu” Gymnasium School, “Kinderakademie” Kindergarten and “Pisicile Aristocrate” Kinndergarten, with the help of the School Inspectorate of Bucharest (ISMB), to organize a charity fair for Christmas, at Verona Garden, for Tessa Serban, a 6-year-old girl who was diagnosed with acute lymphoblastic leukemia.

The initiative of organizing the fair for Christmas gave reasons for many reaction echoes, among which, we will particularly mention one. Mirela Timiras, who took part in the organizing team of the Christmas Fair, decided to be more than only a member of the team and organized, together with the members of Proiectul Tivodar and Journey Pub, a charity concert to sustain this case. All the funds that have been raised as a result of the event being transferred to Tessa’s parents. 

“Proiectul Tivodar” Band held an acoustic concert for Tessa on December 9, 2104, in the welcoming and intimate atmosphere of Journey Pub in Bucharest.

**Fig. 1:**
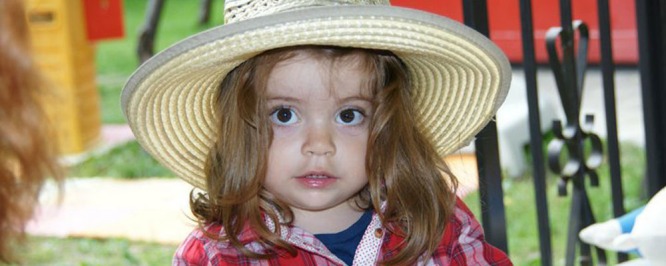
Tessa, a sweet, lovely child

In 2012, the little and sweet Tessa was diagnosed with acute lymphoblastic leukemia. In July 2014, the doctors agreed that Tessa should have access to all the activities, which are characteristic for her age, and so everything seemed to go back to normal. Tessa enrolled in school and enthusiastically started the preparatory level in September 2014. 

**Fig. 2:**
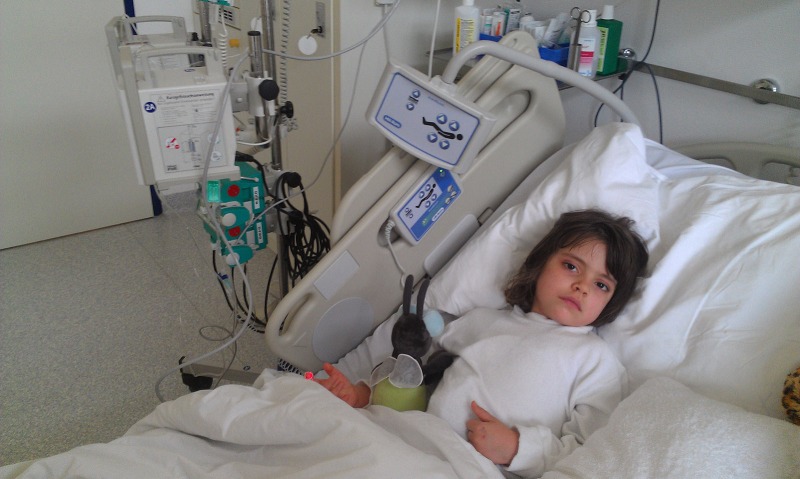
Tessa, at the onset of the disease

In less than 3 months from the treatment (which presupposed daily low chemotherapy sessions), Tessa caught a cold and missed from school, then accused acute, atypical headaches. A complete set of blood and exudate analyses were immediately undergone, without finding any serious sign of disease worsening. While doing an emergency MRI in Romania, it was found that she had a sinusitis that seemed to explain the headaches. Because the term for the first post-treatment clinical investigation was approaching, Tessa went to Vienna for a routine check-up. Here, nothing new was found, her blood analyses were good, the EEG, EKG and CT-scan did not signal any problem, so Tessa was getting ready to come back in Romania. Then, suddenly, three hours before the plane took off; the clinic urgently requested further investigations. The investigations were requested because at a molecular biopsy of the blood, atypical cells were found. Punctures of the bone marrow and lumbar region were immediately done and, in two hours, the unwanted diagnosis was made: her disease relapsed. At that point, the first intensive set of treatment options, during which she received many drugs daily, including two punctures in the spine through which the chemotherapy medicine was injected directly in the cephalorachidian liquid (intrathecal), was undergone. Besides the physical side effects, the psychological ones were overwhelming. At present, Tessa is hardly dealing with the shock. According to the doctors’ prognosis, the risks of the treatment are high, but the healing chances can rise to 80% if a transplant was done. According to the last treatment protocols, ***the transplant is considered compulsory*** for the very rare early isolated disease relapse cases in the Central Nervous System. If the relapse had happened in the bone marrow, the healing would have been less likely. Without a transplant, Tessa’s chances of healing would diminish and the chances of a new relapse would appear. Even from the beginning, the doctors explained that even if they did not find many leukemic cells in the bone marrow so as to diagnose the relapse, their presence, even if in such a low percent (2-3%), which is considered to go in the category of Minimal Residual Disease, negatively influences the prognosis, compared to the case in which the marrow would have been 100% healthy. Moreover, what is very important is the fact that Tessa had a disease relapse too soon, at only 3 months after the treatment was completed. If the disease had relapsed after more than 6 months and the marrow had been 100% clean, probably the allogenic transplant of stem cells would have been avoided.

**Fig. 3:**
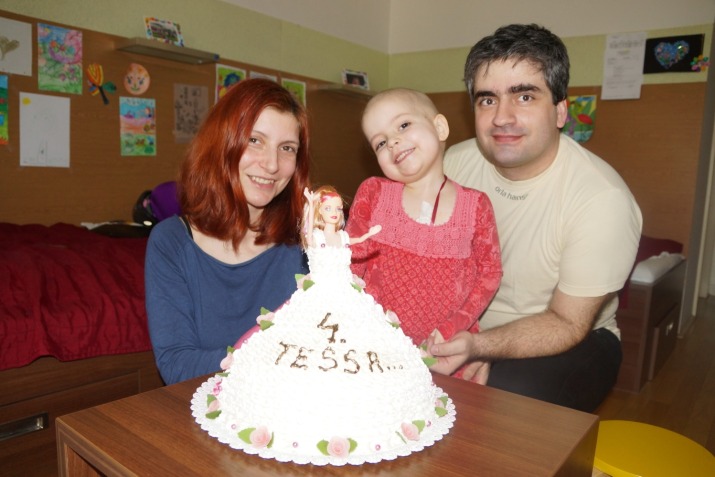
Tessa and her loving parents – 4 years old birthday

“There are many sad things which we could add, but we would rather focus on the positive aspects and the good news that we can share: from a medical point of view, Tessa is answering very well to the treatment; she has already entered the second remission and, at the last punctures of both the bone marrow and the lumbar one, the doctors did not find any trace of the cancerous cells”, Tessa’s wonderful parents affirmed not a very long time ago.

The doctors say there are little chances that a new donor is compatible in such cases, but, in the same time, there are chances to find a donor who is already registered in the international databases. That is why, it is necessary that the ones who wish to save a life by donating stem cells to make an early subscription in the national database. The more the database raises due to the stem cells donors, the bigger the probability of saving lives.

Luckily, Tessa found such a donor. Investigations are still being made in order to assure the full compatibility, but everything seems to be on the right track. 

**Fig. 4:**
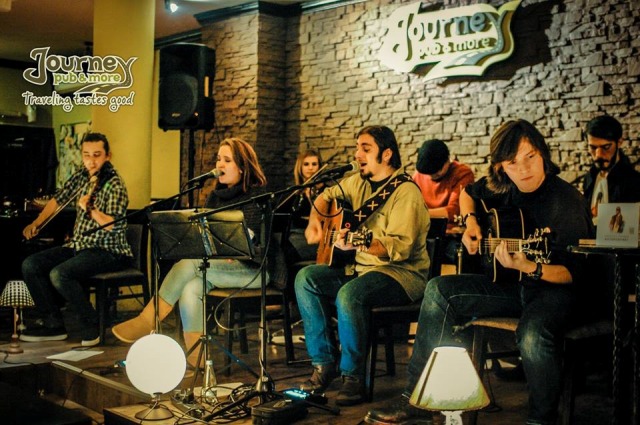
“Proiectul Tivodar” Band

Tessa’s case needs not only responsibility and financial support but also moral support. Such a step was made on December 9, 2014 by the great “ad hoc” team, made up by Mirela Timiras, the members of “Proiectul Tivodar” and the dynamic, receptive and sensitive managerial team of Journey Pub. 

“Proiectul Tivodar” is a young band, already known among the circles of people who enjoy listening to quality music. Their first album “Autoportret” sounds very good, some of songs have already been included in the playlists of many online radios such as 3Net Radio, Trib Radio, Definite Rock, etc. The band was characterized as being the promoter of a music genre that is very close to folk music, also having rock and blue influences.

Other people, rather young in spirit than in age have also contributed to this event, although they were not present, such as two doctors, husband and wife, from “Carol Davila” Clinical Nephrology Hospital, who, although they could not take part, they were beside little Tessa by contributing to the collective charity raise at Journey Pub.

With the feeling that I am not wrong, I can state that I have seen some representatives of the leaders of the students at “Carol Davila” University of Medicine and Pharmacy, in Bucharest, taking part in the welcoming and exceptional atmosphere in Journey Pub.

And, at the end, I could not help noticing the discrete but efficient presence and support of “Carol Davila” Foundation. 

**Dear Tessa, you are not alone.**

**Assoc. Prof. Eng. VL Purcarea**

